# Impact of first ambulation time on unilateral biportal endoscopy in lumbar disc herniation: a systematic review and meta-analysis

**DOI:** 10.1097/JS9.0000000000002686

**Published:** 2025-06-20

**Authors:** Yi Tang, Zhongyi Zhang, Zhouhan Wu, Zhaokai Jin, Yichen Gong, Wen Zhang, Kai Cheng, Jiaju Zhou, Peijian Tong, Taotao Xu, Shuaijie Lv

**Affiliations:** a Orthopedics and Traumatology Medical Center, The First Affiliated Hospital of Zhejiang Chinese Medical University (Orthopedics and Traumatology Hospital of Zhejiang Chinese Medical University), Hangzhou, Zhejiang, China; b Graduate School of Guangxi University of Traditional Chinese Medicine, Nanning, Guangxi, China

**Keywords:** clinical efficacy and complications, lumbar disc herniation, meta-analysis, unilateral bimanual endoscopy

## Abstract

**Background:**

This study aimed to evaluate the effect of early versus late postoperative ambulation on clinical outcomes and complications following unilateral biportal endoscopy (UBE) for lumbar disc herniation.

**Methods:**

A systematic review and meta-analysis was conducted in accordance with PRISMA 2020 guidelines. PubMed, Embase, Web of Science, and Cochrane Library were searched up to February 2025. Eligible studies were grouped based on ambulation timing: early (<24 hours) versus late (≥24 hours). Outcomes included changes in leg pain and back pain measured by the visual analog scale, functional recovery via the Oswestry Disability Index (ΔODI), complication rates, and patient satisfaction (MacNab criteria). A random-effects model was used to pool data and calculate weighted mean differences (WMDs) and 95% confidence intervals (CIs).

**Results:**

Fourteen retrospective studies comprising 715 patients were included (early group: 230; late group: 485). Early ambulation significantly improved leg pain within 3 days (WMD = −6.87; 95% CI: −7.48 to −6.26; *P* < 0.001) and at 6 months (WMD = −7.59; 95% CI: −7.98 to −7.20; *P* < 0.001), both exceeding the minimal clinically important difference. No significant between-group differences were observed for back pain (all time points), functional outcomes (ΔODI), complication rates, or patient satisfaction.

**Conclusions:**

Early ambulation (<24 hours) after UBE is associated with superior short-term relief of leg pain and does not increase postoperative complication risk. However, it offers no advantage in long-term pain, disability, or satisfaction. Early mobilization can be safely recommended as part of postoperative care, with individualized protocols based on patient and surgical factors.

## Introduction

Lumbar disc herniation (LDH), an important type of degenerative disease of the spine, has become one of the leading causes of lumbosacral radicular pain and dysfunction worldwide^[1]^. Epidemiological investigations have shown that LDH has a significant increasing trend, and the age distribution of disease onset has shown forward-shifting characteristics, which has become an important public health problem affecting the health of the working population^[2]^. The disease not only seriously affects patients’ daily life, work, and social activities but also causes considerable social and medical burdens^[3]^. Unilateral bimanual endoscopy (UBE) provides a minimally invasive solution with enhanced visualization and precision and has been widely used in recent years in the clinical treatment of LDH^[4]^. UBE is mostly performed through an interlaminar route that offers proper visualization for most LDH without extensive bone work^[5]^. Compared with traditional open surgery, the UBE technique results in very low intraoperative blood loss and reduces hospitalization time^[6]^. Less musculoskeletal damage helps maintain spinal stability and speed recovery after surgery^[5,7]^.

**HIGHLIGHTS**

- Early ambulation (<24 hours post-unilateral biportal endoscopy [UBE]) significantly improves leg pain relief within 3 days and at 6 months, exceeding minimal clinically important differences.
- No significant differences were observed in back pain relief, functional recovery (Oswestry Disability Index), complication rates, or patient satisfaction between early and late ambulation groups.
- This is the first systematic review comparing UBE postoperative ambulation timing, supporting early mobilization as safe and effective for short-term leg pain improvement.
- Long-term outcomes (12 months) showed convergence between groups, suggesting early ambulation does not compromise long-term recovery.
- Personalized rehabilitation plans (considering age, surgical complexity, and intraoperative factors) are recommended to optimize recovery after UBE.

Unlike other surgical approaches such as open discectomy, microdiscectomy, or full-endoscopic procedures, UBE offers a unique combination of direct visualization, continuous irrigation, and two-portal manipulation, which collectively enable early mobilization while minimizing iatrogenic trauma^[8]^. These technical features make UBE a more homogeneous and appropriate platform for evaluating the influence of first ambulation timing^[9]^. Therefore, this study deliberately focused only on UBE-based interventions to avoid the heterogeneity and confounding factors associated with broader inclusion of disparate surgical techniques for LDH. Early mobilization has been increasingly integrated into enhanced recovery after surgery (ERAS) protocols in spinal procedures, with studies suggesting benefits in reducing postoperative complications, shortening length of stay, and promoting functional recovery following lumbar fusion or discectomy^[10]^. However, current evidence remains largely limited to traditional or instrumented spine surgeries, and few studies have specifically addressed optimal ambulation timing following UBE.

However, the optimal time of first ambulation for patients after UBE is not yet clinically standardized. The timing of the first postoperative ambulation is directly related to the patient’s surgical outcome and recovery process^[11]^. Early activity may help promote the recovery of gastrointestinal function, prevent deep vein thrombosis, and increase patient confidence, but at the same time, it may also increase the risk of complications such as cerebrospinal-fluid (CSF) leakage caused by transient rises in intraspinal pressure when an unintended dural breach is present^[12,13]^. Because UBE for LDH is a decompression-only technique that involves no internal instrumentation, the previously mentioned concern regarding hardware loosening is not applicable and has been removed. Although early ambulation is theoretically more likely to influence short-term physiological indicators such as length of stay, opioid consumption, and gastrointestinal recovery, most of the currently available studies on UBE fail to report these outcomes in a standardized manner. As a result, this study focused on patient-reported outcomes, which were the most consistently reported and extractable endpoints across the included literature^[14]^. Therefore, the aim of this study was to systematically collect and analyze strong evidence that the time to first postoperative ambulation influences outcomes related to the efficacy of UBE in the treatment of LDH, thereby providing relevant evidence-based insights into the postoperative management of LDH.

## Methods and materials

This systematic review was conducted in accordance with the Preferred Reporting Items for Systematic Reviews and Meta-Analyses (PRISMA) 2020 statement^[15]^ and was reported in line with Assessing the Methodological Quality of Systematic Reviews Guidelines^[16]^. Our meta-analysis review protocols were registered on International Prospective Register of Systematic Reviews. Reporting of this study complies with the Transparency In The Reporting of Artificial Intelligence (TITAN) Guidelines for transparent scholarly communication^[17]^, ensuring full methodological disclosure despite the absence of artificial intelligence (AI) utilization.

### Search strategy

To perform a comprehensive search strategy, we systematically searched for relevant studies published in electronic databases, including PubMed, Web of Science, Cochrane Clinical Trials, and Embase, and supplemented the literature through Google. Searches were conducted by combining subject terms and free words. The search terms were adjusted to the specific database. The final search timeframe was from database construction to February 22, 2025. The specific search strategy is shown in Appendix 1, available at: http://links.lww.com/JS9/E429.

In addition, we performed a manual check through the reference list for identification.

### Inclusion and exclusion criteria

The criteria for inclusion in our meta-analysis were as follows: (1) all types of clinical studies other than case reports were included, including prospective, retrospective cross-sectional, cohort, and case-control studies; (2) patients with definite clinical symptoms and a diagnosis of LDH confirmed by magnetic resonance or X-ray or diagnostic criteria reported in the original article; (3) all patients with LDH who underwent surgical treatment with UBE; and (4) adequate data, including time to first postoperative ambulation, postoperative clinical or functional outcomes, and complication information, were needed.

Studies were excluded if they met any of the following criteria: (1) studies that were not peer-reviewed or lacked relevant data, including but not limited to case series, technical notes, conference reports, and review articles; (2) studies that published duplicate data or contained incomplete statistical data; (3) studies published in languages other than English or Chinese; (4) fusion surgery was performed, or the site of surgery was the cervical spine.

### Data extraction

All titles and abstracts of the reports were carefully screened and then thoroughly assessed for eligibility of full-text studies on the basis of established inclusion and exclusion criteria. Reports deemed eligible were thoroughly reviewed, and relevant data were systematically recorded. Prior to the formal commencement of data extraction, both researchers were given uniform training to independently extract data from five literature articles and calibrate them for consistency. The two researchers then independently screened the literature, extracted the data, and cross-checked them against the inclusion and exclusion criteria. Disagreements, if any, were discussed and resolved, and a third investigator was consulted if necessary. The kappa index was reported to cue researcher concordance^[18]^. If the information in the study was incomplete, the author was contacted for more information. A pre-designed data-extraction form was used to extract information. The main information extracted from the data included (1) general information about the included studies, including title, authors, and year of publication; (2) characteristics of the studies, including study area, sample size, age, duration of surgery, and duration of follow-up; (3) data on relevant outcome indicators and outcome measures; and (4) data related to risk-of-bias assessment.

## Quality assessment

The methodological index for non-randomized studies (MINORS) was used to assess the risk of bias^[19]^. Since only single-arm outcomes were assessed in this study, only the first eight items were used for assessment. Two investigators independently assessed the risk of bias. Disagreements, if any, were discussed and resolved, and a third investigator was consulted if necessary.

### Outcome indictor

The studies were categorized into early (ambulation initiated within the first 24 hours) and late (after 24 hours) groups, in keeping with ERAS guidance for spinal procedures, which designates mobilization on the day of surgery or within 24 hours as “early” mobilization^[20]^; moreover, 9 of the 14 studies included in this review employed the identical 24-hour threshold, thereby enhancing cross-study comparability.

The studies were categorized into early (<24 hours) and late (≥24 hours) groups on the basis of the time of first postoperative walking. The following information was extracted: (1) Pain relief: The change in the visual analog scale score for leg pain and back pain within 3 days, 3 months, 6 months, and 12 months postoperatively compared with the preoperative value. (2) Functional recovery: the value of the change in the Oswestry dysfunction index (ODI) score (ΔODI) within 3 days, 3 months, 6 months, and 12 months postoperatively compared with the preoperative value. (3) Complication rate; (4) patient satisfaction. The minimum clinically important difference (MCID) was set at 12.8 for the ODI, 1.2 for the VAS score for back pain, and 1.6 for leg pain^[21]^.

### Statistical analysis

Analyses were conducted via the statistical software Stata (Stata Corp, College Station, TX, USA) version 17.0. Since the incidence of the original data is not within the range of 30%–70%, the data were transformed via the double-arcsine transformation with metaprop, and then statistical pooling was performed^[19]^. *I*^2^ was used to test for heterogeneity in the included studies. According to the Cochrane Handbook, *I*^2^ > 50% was considered heterogeneity, and a random-effects model was selected for combinatorial statistical analysis; otherwise, a fixed-effects model was selected for data processing and analysis.

In addition, to further explore the potential sources of heterogeneity, we performed a meta-regression analysis using six prespecified covariates: male proportion (≥50%), age (mean >50 years), operative time (>80 minutes), sample size (≥100), publication year (≥2024), and number of operated segments (≥3). The meta-regression results were summarized in Table 1, and variables showing statistically significant associations (e.g. *P* < 0.05) were highlighted and interpreted in the Discussion section.

**Table 1:** The results of meta-regression

Outcome indicators	Male proportion (≥50% male)	Age >50 years	Operative duration >80 min	Sample size ≥100	Publication year ≥2024	Operated segments ≥3
Postoperative leg VAS	*P* = 0.06	*P* = 0.80	*P* = 0.17	*P* = 0.71	*P* = 0.15	*P* = 0.43
Postoperative back VAS	*P* = 0.27	*P* = 0.15	*P* = 0.93	*P* = 0.27	*P* = 0.15	*P* = 0.27
Postoperative ODI	*P* = 0.70	*P* = 0.07	*P* = 0.44	*P* = 0.02*	*P* = 0.78	*P* = 0.44
3-month leg VAS	*P* = 0.57	*P* = 0.29	*P* = 0.78	*P* = 0.60	*P* = 0.25	*P* = 0.74
3-month back VAS	*P* = 0.36	*P* = 0.02*	*P* = 0.43	*P* = 0.10	*P* = 0.42	*P* = 0.36
3-month ODI	*P* = 0.03*	*P* = 0.28	*P* = 0.32	*P* = 0.23	*P* = 0.05*	*P* = 0.04*
6-month leg VAS	*P* = 0.80	*P* = 0.60	*P* = 0.80	*P* = 0.91	*P* = 0.10	*P* = 0.80
6-month back VAS	NA	*P* = 0.75	NA	NA	*P* = 0.37	NA
6-month ODI	*P* = 0.51	*P* = 0.10	*P* = 0.51	*P* = 0.29	*P* = 0.89	*P* = 0.29
12-month leg VAS	*P* = 0.73	*P* = 0.83	*P* = 0.55	*P* = 0.60	*P* = 0.38	*P* = 0.83
12-month back VAS	*P* = 0.74	NA	*P* = 0.97	NA	*P* = 0.24	*P* = 0.74
12-month ODI	*P* = 0.70	*P* = 0.19	*P* = 0.81	*P* = 0.69	*P* = 0.79	*P* = 0.69
Complications	*P* = 0.85	*P* = 0.79	*P* = 0.68	*P* = 0.81	*P* = 0.75	*P* = 0.95
Clinical satisfaction (MacNab criteria)	*P* = 0.79	*P* = 0.46	*P* = 0.87	*P* = 0.24	*P* = 0.67	*P* = 0.70

^*^ *P* < 0.05.

### Sensitivity analysis

We used sensitivity analysis to evaluate the robustness of the results. During the stage of selecting statistical methods, we applied the random-effects model or the fixed-effects model to assess the overall effect. Additionally, by sequentially excluding studies one by one and then reperformed the meta-analysis to evaluate the robustness of the results. After excluding specific studies, significant changes in the combined effect size indicated a considerable influence on the overall result.

### Publication bias

Although the Cochrane Handbook suggests applying publication bias to outcomes reported in more than 10 studies, we conducted Egger’s test because each included outcome involved ≥5 studies and the total number of participants exceeded 250, a threshold under which the test has been shown to retain acceptable statistical power and type I error rates^[22,23]^. The test yielded *P* > 0.05, indicating no significant small-study effects.

### Certainty assessment

The evidence level was evaluated via the GRADE profile software version 3.6, in accordance with the Grading of Recommendations, Assessment, Development and Evaluation framework. The assessment considered five factors for downgrading the evidence (risk of bias, inconsistency, indirectness, imprecision, publication bias) and three factors for upgrading the evidence (large effect, plausible confounding that could alter the effect, dose-response gradient). Based on this evaluation, the evidence quality was categorized as high, moderate, low, or very low.

## Results

### Literature search results

**Figure 1.**
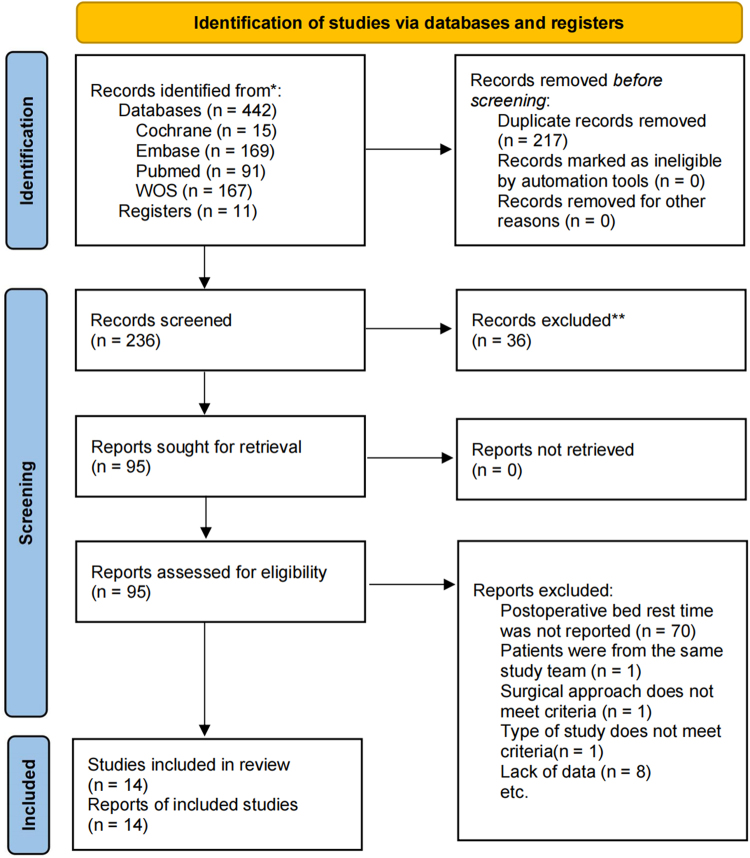
PRISMA flowchart of the literature search.

A total of 442 articles were initially collected in this study: 91 from PubMed, 169 from Embase, 362 from Web of Science, 15 from the Cochrane Library, and 11 records from clinical trial registries. After excluding duplicate entries, 236 studies remained. Then the titles and abstracts were reviewed, and 95 articles were selected for further analysis. The study by Zuo *et al*^[24]^ was excluded because it failed to rule out duplicate patients. More studies were excluded because they failed to report the first ambulation time. A total of 14 studies were included in the meta-analysis, including 6 in English^[25–30]^ and 8 in Chinese^[31–39]^. The kappa score of the review process was 0.88, indicating an excellent level of consistency among the researchers. Screening procedures and corresponding results are detailed in the PRISMA flowchart (Fig. 1).

### Study characteristics and quality assessment

Table 2 describes the characteristics and details of the included studies. The 14 studies included a total of 715 patients. The early group consisted of four studies with a total of 230 patients. The late group included 10 studies with a total of 485 patients. All studies were retrospective. Most of the studies were from China, with only one study from Vietnam. Table 3 provides a summary of the quality assessment of non-randomized studies based on the MINORS scale.

**Table 2:** Characteristics of included studies

Study	Study design	Country	Sample size	Age	Surgical segment	Gender (male/female)	First ambulation time	Postoperative hospital stay (day)	Follow-up duration (month)	Complications	MacNab
Chen 2024	Retrospective	China	30	41.2 ± 15	L3-S1	12/8	6 hours	6.7 ± 1.9	12	2	26
Su 2025	Retrospective	China	20	50.4 ± 14	L3-S1	9/1	2 days	NA	6	1	19
Li 2024	Retrospective	China	109	53.3 ± 8.2	L3-S1	47/62	2 days	5.3 ± 3.7	19.6 ± 7.2	10	97
Zhu 2022	Retrospective	China	19	39.1 ± 7.75	L4-S1	2/7	3 days	NA	8.2 ± 1	0	18
Wang 2022	Retrospective	China	101	37.9 ± 13	L3-S1	60/41	1 day	5.23 ± 2.84	6	7	NA
Shen 2024	Retrospective	China	54	38.7 ± 9.2	L4-S1	32/22	2.4 ± 0.57 hours	3.8 ± 0.61	10.69 ± 2.49	0	52
Zhu 2023	Retrospective	China	32	54.5 ± 13.2	L4-S1	17/15	12 hours	4.9 ± 1.2	6	0	30
Xu 2023	Retrospective	China	17	59.2 ± 6.5	L4-S1	9/8	1 day	2 ± 0.5	10–24	7	17
Chang 2022	Retrospective	China	42	36.3 ± 12.2	L4-S1	8/34	2.9 ± 0.9 days	4.7 ± 1.8	12	6	39
Wu 2024	Retrospective	China	31	58.5 ± 13.2	L4-S1	16/15	1 day	4.5 ± 2.6	8.6 ± 2.9	1	28
Zhu 2023	Retrospective	China	100	54.44 ± 13.37	Not reported	54/46	1 day	NA	12	NA	75
Duong 2023	Retrospective	Vietnam	46	49.5 ± 11.8	L2-S1	18/28	1 day	4.3 ± 2.1	1–6	3	44
Qin 2025	Retrospective	China	72	73.2 ± 8.5	L3-S1	43/29	2–6 hours	NA	14.2 ± 1.9	3	65
Zuo 2022	Retrospective	China	42	45.57 ± 11.15	L5-S1	23/19	4 hours	6.88 ± 1.85	12	1	NA

NA, not applicable.

**Table 3:** MINORS scale for assessing the quality of studies included in the meta-analysis

Study	A clearly stated aim	Inclusion of consecutive patients	Prospective collection of data	Endpoints appropriate to the aim of the study	Unbiased assessment of the study endpoint	Follow-up period appropriate to the aim of the study	Loss to follow up less than 5%	Prospective calculation of the study size	Total
Chen 2024	1	2	2	2	0	1	2	0	10
Su 2025	0	2	2	2	0	1	2	0	9
Li 2024	1	2	2	2	0	2	2	0	11
Zhu 2022	2	2	2	1	0	1	2	0	10
Wang 2022	2	2	2	1	0	1	2	0	10
Shen 2024	0	2	2	2	0	1	2	0	9
Zhu 2023	1	2	2	2	0	1	2	0	10
Xu 2023	0	2	2	2	0	2	2	0	10
Chang 2022	2	2	2	2	0	2	2	0	12
Wu 2024	2	2	2	2	0	1	2	0	11
Zhu 2023	2	2	2	2	0	2	2	0	12
Duong 2023	2	2	2	2	0	0	2	0	10
Qin 2025	2	2	2	2	0	2	2	0	12
Zuo 2022	2	2	2	2	0	2	2	0	12

### Meta-analysis results

**Figure 2.**
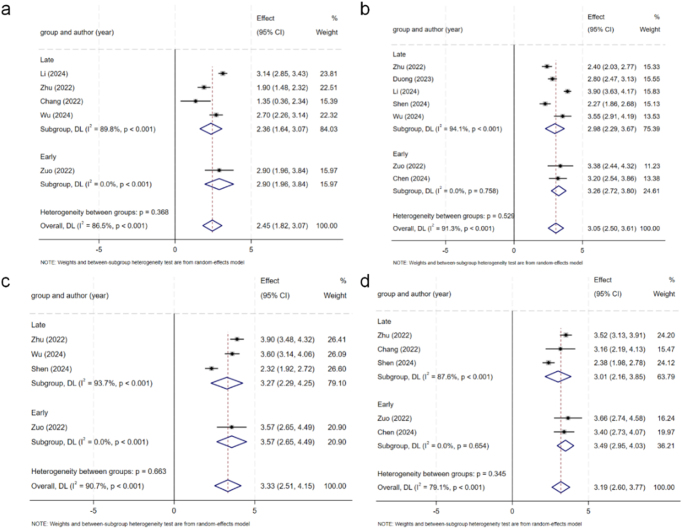
Forest plot of the ΔVAS score for back.

**Figure 3.**
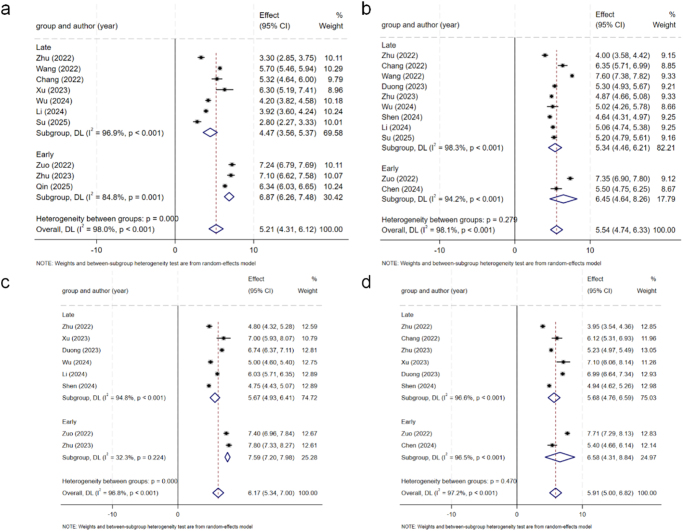
Forest plot of the ΔVAS score for leg pain.

**Figure 4.**
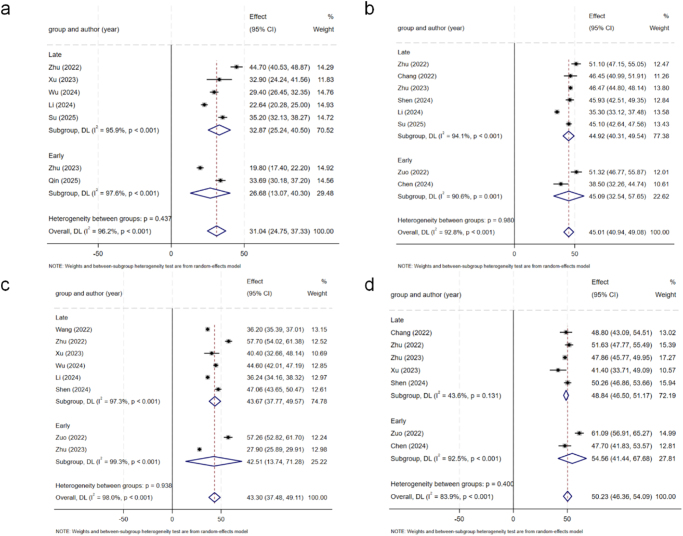
Forest plot of the ΔODI scores.

**Figure 5.**
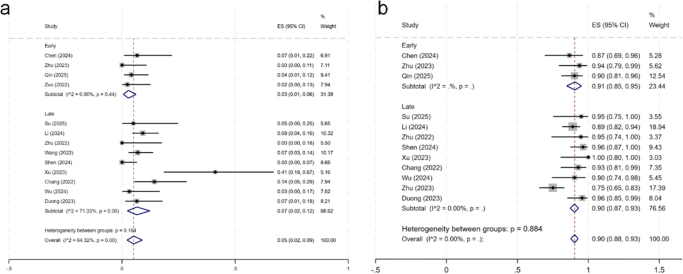
Forest plot of the complication rate and MacNab criteria.

Table 4 shows the comparison results in detail. Most of the comparisons were extremely heterogeneous, so a random effects model was used for analysis. The *I*^2^ values for each outcome are presented in Table 4 to quantify the heterogeneity, with several comparisons exceeding the 75% threshold, indicating substantial inconsistency. In terms of the VAS scores for back pain (Fig. 2), there were no significant differences between the early and late groups at any time point (*P* > 0.05). The VAS score for leg pain (Fig. 3) revealed that the score of the early group was significantly greater than that of the late group (*P* < 0.001) within 3 days and 6 months after surgery, and the improvement was greater than the MCID. However, there was no significant difference between the two groups at 3 and 12 months after surgery (*P* > 0.05). In terms of the ODI scores (Fig. 4), there were no significant differences between the early group and the late group at any time point (*P* > 0.05). There were no significant differences in the final complication rate or MacNab criteria between the early and late groups (Fig. 5) (*P* > 0.05).

**Table 4:** Detailed results of the meta-analysis

Item	Time point	Number of study	Early group			Late group			Heterogeneity (*I*^2^, *P*)	Heterogeneity between groups *P*
			*n*	WMD (95% CI)	Heterogeneity (*I*2, *P*)	*n*	WMD (95% CI)	Heterogeneity (*I*^2^, *P*)		
ΔVAS scores for back pain	Within postoperative 3 days	5	42	2.90 (1.96–3.84)	NA^a^	201	2.36 (1.64–3.07)	89.8%–<0.001	86.5%, <0.001	0.368
	3 months	7	72	3.26 (2.72–3.80)	0.0%, 0.758	270	2.98 (2.29–3.67)	94.1%, <0.001	91.3%, <0.001	0.529
	6 months	4	42	3.57 (2.65–4.49)	NA^a^	104	3.27 (2.29–4.25)	93.7%, <0.001	90.7%, <0.001	0.663
	12 months	5	72	3.49 (2.95–4.03)	0.0%, 0.654	205	3.01 (2.16–3.85)	87.6%, <0.001	79.1%, <0.001	0.345
ΔVAS scores for leg pain	Within postoperative 3 days	10	146	6.87 (6.26–7.48)	84.8%, 0.001	341	4.47 (3.56–5.37)	96.9%, <0.001	98.0%, <0.001	<0.001
	3 months	10	72	6.45 (4.64–8.26)	94.2%, <0.001	510	5.34 (4.46–6.21)	98.3%, <0.001	98.1%, <0.001	0.279
	6 months	8	74	7.59 (7.20–7.98)	32.3%, 0.224	290	5.67 (4.93–6.41)	98.4%, <0.001	96.8%, <0.001	<0.001
	12 months	8	72	6.58 (4.31–8.84)	96.5%, <0.001	205	5.68 (4.76–6.59)	96.6%, <0.001	97.2%, <0.001	0.47
ΔODI values	Within postoperative 3 days	7	114	26.68 (13.07–40.30)	97.6%, <0.001	188	32.87 (25.24–40.50)	95.9%, <0.001	96.2%, <0.001	0.437
	3 months	8	72	45.09 (32.54–57.65)	90.6%, <0.001	321	44.92 (40.31–49.54)	94.1%, <0.001	92.8%, <0.001	0.98
	6 months	9	74	42.51 (13.74–71.28)	99.3%, <0.001	244	43.67 (37.77–49.57)	97.3%, <0.001	98.0%, <0.001	0.938
	12 months	8	72	54.56 (41.44–67.68)	92.5%, <0.001	324	48.84 (46.50–51.17)	43.6%, 0.131	83.9%, <0.001	0.4
Complication rate	NA	13	176	0.03 (0.01–0.06)	0.00%, 0.44	439	0.07 (0.02–0.12)	71.3%, <0.001	64.3%, <0.001	0.164
MacNab criteria		12	134	0.91 (0.85–0.95)	NA^b^	438	0.90 (0.87–0.93)	NA^b^	NA	0.884

NA, not available; ΔVAS scores, the reduction in VAS score from preoperative to postoperative periods; ΔODI values, the reduction in ODI values from preoperative to postoperative periods.

^a^ Only one study was included.

^b^ *I*^2^ = 0.

To further investigate the potential sources of heterogeneity, we performed a meta-regression analysis. The results, presented in Table 1, revealed several statistically significant associations. Specifically, sample size ≥100 was significantly associated with postoperative ODI (*P* = 0.02); age >50 years was significantly associated with 3-month back pain VAS (*P* = 0.02); and for 3-month ODI, male proportion ≥50%, publication year ≥2024, and operated segments ≥3 were all significantly associated with the effect size (*P* < 0.05). These findings suggest that patient demographics, study scale, and surgical extent may partially explain the heterogeneity observed across studies.

### Sensitivity analysis and publication bias

The sensitive analysis demonstrated that the effect sizes remained largely consistent upon the exclusion of individual studies, thereby indicating the robustness of the results. Egger’s test was used to assess the risk of publication bias. The *P* values of the VAS scores and ODI scores at different times are shown in Table 5. There was no publication bias in almost all outcome variables, including surgical complications (*P*= 0.601) and the MacNab criteria (*P*= 0.05).

**Table 5:** The *P* value of Egger’s test for the vast majority of surgical outcome variables

Time point	ΔVAS scores for low back pain	ΔVAS scores for leg pain	ΔODI values
Postoperative within 3 days	0.36	0.841	0.139
Postoperative 3 months	0.746	0.589	0.657
Postoperative 6 months	0.756	0.451	0.134
Postoperative 12 months	0.589	0.573	0.836

ΔVAS scores, the reduction in VAS score from preoperative to postoperative periods; ΔODI values, the reduction in ODI values from preoperative to postoperative periods.

### Certainty assessment

In most comparisons, the quality of evidence is found to be very low due to inconsistency and imprecision. In the MacNab criteria, the quality of evidence is regarded as moderate (Supplemental Digital Content Appendix 2, available at: http://links.lww.com/JS9/E429).

## Discussion

This meta-analysis of 14 studies involving 715 patients aimed to evaluate the impact of early ambulation on clinical outcomes following UBE for LDH. Three key findings were identified. First, early ambulation significantly reduced leg pain within 3 days and at 6 months postoperatively compared to late ambulation, with effect sizes exceeding the MCID, indicating a meaningful short-term analgesic effect. However, no differences were observed in back pain (VAS-back) or functional recovery (ODI) at any follow-up point. Second, early ambulation did not increase the overall complication rate and showed no benefit in patient-reported satisfaction. Final complication rates and MacNab criteria scores were comparable between groups, suggesting that early mobilization is safe but does not confer additional advantages in global subjective outcomes. Third, meta-regression analysis revealed that several patient and study characteristics influenced outcomes. Sample size was significantly associated with postoperative ODI, age with 3-month back pain scores, and male proportion, publication year, and number of operated segments with 3-month ODI. These findings suggest that demographic and procedural factors may modify the relationship between ambulation timing and recovery. Overall, early ambulation after UBE appears to offer short-term pain benefits without increasing complication risk, although its broader functional impact may be limited and context-dependent.

Pain and functional impairment, particularly leg pain, back pain, and mobility restriction, are the most common and debilitating symptoms in patients with LDH, and are therefore critical indicators for assessing postoperative recovery^[40]^. In this study, early ambulation significantly alleviated leg pain in the short term but showed no measurable benefit on back pain or functional improvement, prompting further consideration of the underlying mechanisms. This pattern is partially consistent with prior findings. Several clinical studies have reported that early mobilization after lumbar surgery can enhance circulation, reduce nerve root edema, and promote neural decompression, resulting in faster pain relief – particularly in radicular symptoms. For example, Park *et al*^[41]^ observed transient improvements in lower limb symptoms with early walking after endoscopic discectomy, while no significant functional gains were noted^[41]^. Our findings reinforce this short-term effect on leg pain, but similarly demonstrate limited impact on broader functional outcomes or axial pain. The selective improvement in leg pain may be attributed to early reductions in mechanical nerve root compression and inflammatory mediator accumulation. Early ambulation may enhance venous and lymphatic drainage, thereby reducing perineural congestion and alleviating acute nociceptive signaling along the sciatic pathway^[42]^. In contrast, back pain in LDH patients often arises from paraspinal muscle dissection, joint stress, or discogenic degeneration, which are less directly affected by mobilization timing^[10]^. Moreover, functional recovery, as reflected by the ODI, is a multidimensional construct encompassing physical endurance, coordination, and psychological adaptation – domains unlikely to improve solely through early mobilization^[43]^. The lack of sustained benefit beyond 6 months may reflect the natural course of disc healing and neuroplastic adaptation. As acute inflammation resolves and the body progresses toward structural remodeling, the initial advantages of early ambulation become less influential^[44]^. Long-term outcomes are more likely to depend on factors such as core stability, rehabilitation intensity, and structural degeneration rather than ambulation timing alone.

Consistent with the absence of improvement in functional scores, our findings also revealed no significant difference between early and late ambulation groups in subjective recovery as measured by the MacNab criteria. This lack of enhancement in patient-reported global outcomes suggests that early ambulation alone may be insufficient to influence broader perceptions of recovery, which are typically shaped by multifactorial domains such as physical capacity, psychosocial adaptation, and rehabilitation engagement. These elements often extend beyond the scope of immediate postoperative mobilization. Although early mobilization is frequently encouraged in postoperative protocols, concerns persist regarding its potential to increase complications, including CSF leakage, wound problems, and neurological deterioration – particularly in the context of endoscopic spine surgery where the surgical field is narrow and dural integrity is vulnerable. However, our analysis found no significant difference in the overall complication rates between early and late ambulation groups, supporting the safety of initiating early mobilization following UBE procedures. This finding is consistent with previous studies on endoscopic lumbar surgery. For instance, a prospective cohort by Wu *et al*^[45]^ reported comparable complication profiles regardless of ambulation timing, and similar conclusions were drawn in ERAS-based spine surgery trials. Importantly, UBE is a decompression-only technique without instrumentation, and its biportal design allows for continuous irrigation and minimal muscle disruption, thereby reducing the likelihood of structural instability or delayed wound healing – key contributors to postoperative complications^[9]^. Additionally, controlled early mobilization may actually enhance tissue perfusion and reduce venous stasis, indirectly lowering the risk of infection and thromboembolic events. Collectively, these results suggest that early ambulation is a safe postoperative strategy in the context of UBE, with no increase in adverse events, though its benefit in improving perceived functional recovery remains limited.

Given the substantial statistical heterogeneity observed across multiple outcome measures, we conducted a meta-regression analysis to explore potential sources of variability. The analysis identified several significant covariates that may have moderated the relationship between ambulation timing and postoperative outcomes. Specifically, larger sample sizes (≥100) were significantly associated with greater postoperative ODI improvements, suggesting that well-powered studies may better capture subtle functional differences. Older age (>50 years) was linked to higher back pain VAS scores at 3 months, which aligns with known age-related impairments in spinal recovery, including delayed neural remodeling, decreased muscle regeneration, and reduced disc hydration. Furthermore, studies with a higher proportion of male patients, more recent publication years (≥2024), and more extensive surgical involvement (≥3 decompressed segments) were significantly associated with ODI effect sizes at 3 months. These findings likely reflect evolving surgical techniques, reporting standards, and sex-related differences in pain perception and recovery behavior. These results underscore the complexity of evaluating postoperative recovery and suggest that patient demographics, surgical burden, and study design may significantly influence the observed efficacy of early ambulation. Future trials should consider stratified analyses based on these covariates to improve precision and generalizability.

The findings of this meta-analysis provide important guidance for postoperative management following UBE for LDH. Specifically, the demonstrated short-term analgesic benefit and absence of increased complication risk support the incorporation of early ambulation – within 24 hours – into standard enhanced recovery protocols. Given that early mobilization did not compromise safety and may facilitate earlier pain control, its adoption could improve patient comfort, reduce dependency on opioids, and potentially shorten hospital stays when combined with comprehensive rehabilitation. However, the lack of significant effects on long-term functional outcomes and global patient satisfaction indicates that early ambulation should not be viewed as a standalone intervention. Instead, it should be integrated into multimodal recovery strategies that include structured physical therapy, patient education, and individualized rehabilitation planning. Moreover, the moderating effects identified in meta-regression suggest that tailored ambulation protocols based on patient age, surgical complexity, and baseline functional status may further optimize outcomes. In clinical practice, these findings encourage early mobilization after UBE as a safe and potentially beneficial component of perioperative care, while emphasizing the need for broader, multidisciplinary approaches to achieve sustained functional recovery.

This study has notable strengths. It is the first meta-analysis to specifically assess the effect of ambulation timing following UBE for LDH, addressing a clinically important but previously underexplored aspect of postoperative management. Furthermore, by incorporating meta-regression, we were able to identify key moderators – such as age, sample size, and surgical extent – that may influence treatment effects, thereby offering insights beyond pooled estimates. While this study did not employ AI tools, we followed the TITAN Guidelines to ensure maximum transparency in data presentation and analytical processes. Nonetheless, several limitations must be acknowledged. First, all included studies were retrospective in design and lacked prospective or randomized control, rendering them highly susceptible to selection bias and information bias. In particular, patients who ambulated earlier may have had better baseline physical function, lower perioperative risk, or higher motivation, all of which could influence outcomes independent of the intervention itself. Second, the average follow-up duration of 12 months was inadequate to capture long-term complications such as adjacent segment degeneration or reoperation, particularly in older patients or those with multilevel decompression. Third, variability in the definitions of “early” and “late” ambulation, as well as in rehabilitation protocols, limited comparability across studies and may have contributed to the high statistical heterogeneity observed. Additionally, key short-term physiological outcomes – such as length of stay, opioid use, and pulmonary or gastrointestinal recovery – were poorly reported and thus excluded from this analysis, despite their relevance to early ambulation. Lastly, most studies were conducted in East Asian populations and excluded fusion or cervical procedures, limiting the external validity of our findings. Future high-quality randomized controlled trials with longer follow-up durations, standardized definitions of ambulation timing, and comprehensive outcome reporting – including both objective physiological indicators and patient-reported metrics – are needed to validate and extend these findings. Stratified analyses based on age, sex, surgical burden, and baseline functional status may further inform tailored rehabilitation strategies in UBE patients.

## Conclusion

This meta-analysis demonstrated that early ambulation (<24 hours) following UBE for LDH is safe and provides modest short-term relief of leg pain, without increasing complication rates or improving long-term functional outcomes. No significant differences were observed in back pain, disability scores, or patient satisfaction compared to later ambulation (≥24 hours). Given the minimally invasive nature of UBE, early mobilization may be encouraged as part of enhanced recovery protocols. However, its application should be individualized based on patient age, baseline function, and surgical complexity. Further prospective studies are warranted to confirm these findings and optimize ambulation strategies.

## Data Availability

The data of this study is publicly available and can be obtained upon request from the corresponding author.

## Research registration unique identifying number (UIN)

CRD420251000960.
